# Vaping and mental health disorders among medical trainees

**DOI:** 10.31744/einstein_journal/2026AO2193

**Published:** 2026-09-18

**Authors:** Fernanda Gushken, Luiza Helena Degani-Costa, Fernando Pereira Bruno, Claudia Szlejf, Ana Bresser Tokeshi, Yasmin Fakhari Tehrani, Daniel Kaufman, Kandipudi K L Prasad, Pentapati SS Kumar, Limalemla Jamir, Matthew GK Benesch, Morag G Ryan, Hardeep Lotay, Jonathan P Fuld, Thiago Marques Fidalgo

**Affiliations:** 1 Department of Psychiatry University of Miami Jackson Health System Miami United States Department of Psychiatry, University of Miami/Jackson Health System, Miami, United States.; 2 Faculdade Israelita de Ciências da Saúde Albert Einstein Hospital Israelita Albert Einstein São Paulo SP Brazil Faculdade Israelita de Ciências da Saúde Albert Einstein, Hospital Israelita Albert Einstein, São Paulo, SP, Brazil.; 3 Touro College of Osteopathic Medicine New York Middletown United States Touro College of Osteopathic Medicine, New York, Middletown, United States.; 4 Department of Big Data Hospital Israelita Albert Einstein São Paulo SP Brazil Department of Big Data, Hospital Israelita Albert Einstein, São Paulo, SP, Brazil.; 5 Department of Psychiatry Escola Paulista de Medicina Universidade Federal de São Paulo São Paulo SP Brazil Department of Psychiatry, Escola Paulista de Medicina, Universidade Federal de São Paulo, São Paulo, SP, Brazil.; 6 Andhra Medical College Vishakhapatnam India Andhra Medical College, Vishakhapatnam, India.; 7 All India Institute of Medical Sciences Mangalagiri India All India Institute of Medical Sciences, Mangalagiri, India.; 8 All India Institute of Medical Sciences Guwahati India All India Institute of Medical Sciences, Guwahati, India.; 9 Memorial University of Newfoundland Newfoundland and Labrador Canada Memorial University of Newfoundland, Newfoundland and Labrador, Canada.; 10 University of Cambridge Cambridge United Kingdom University of Cambridge, Cambridge, United Kingdom.

**Keywords:** Electronic nicotine delivery systems, Vaping, Substance-related disorders, Mental health, Mental disorders, Students medical

## Abstract

**Objective:**

Electronic cigarettes (e-cigarettes) have gained popularity among young people and, like tobacco smoking, may be associated with mental health disorders. In this secondary analysis of the Global Vaping Study, we aimed to investigate the association between vaping dependence and mental health, including anxiety, depression, and substance use disorders.

**Methods:**

The Global Vaping Study was a multinational web-based survey conducted between October 2020 and November 2021 in Brazil (BR), the United States (US), and India (IN), investigating patterns of use, knowledge, and attitudes of medical trainees toward e-cigarettes and hookah. This study’s primary outcome was vaping dependence, defined as the presence of at least two symptoms from the Hooked on Nicotine Questionnaire. Depression, anxiety, and alcohol use disorder were measured with validated questionnaires, whereas other substances were measured using adapted National Survey questions. A mixed effects model was employed to evaluate associations with mental health and substance use.

**Results:**

Of 6,160 respondents from BR and US, 946 (15.3%) were active e-cigarette users (BR=612, US=334). Among users, 534 (59.6%) reported e-cigarette dependence. Psychiatric treatment in the previous year (OR=1.56, 95%CI=1.10–2.22) and male gender (OR=1.46, 95%CI=1.05–2.04) were linked with vaping dependence.

**Conclusion:**

This study reveals a high prevalence of vaping dependence among medical trainees, particularly among males and those with prior psychiatric treatment. Coordinated efforts involving medical schools, healthcare providers, and regulatory bodies may support education, population-level screening, and referral strategies to address vaping dependence without replacing clinical care.

## Highlights

■ The prevalence of e-cigarette dependence among medical trainees who were active vape users was 59.6%.

■ Male gender and recent psychiatric treatment were associated with higher vaping dependence.

■ A holistic assessment of vaping users may help identify broader mental health risks.

## In Brief

High prevalence of vaping dependence was observed among medical trainees. Dependence was independently associated with prior psychiatric treatment and male gender, suggesting vaping may signal broader mental health vulnerability and highlighting the need for screening and prevention strategies.

## Graphical abstract

**Figure:**
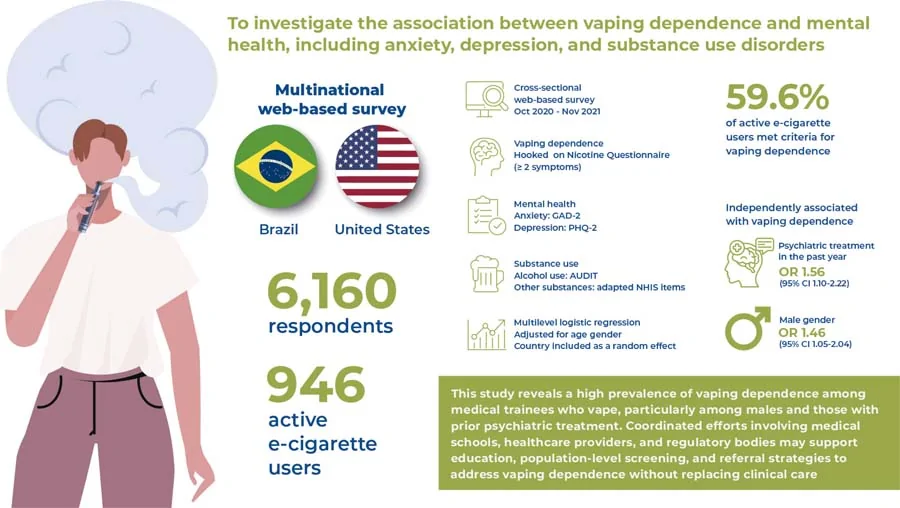

## INTRODUCTION

The popularity of electronic nicotine delivery systems, commonly referred to as electronic cigarettes (e-cigarettes), has surged in recent decades, contributing to the widespread use of vaping devices. However, this rise in popularity has also led to misconceptions about their safety. A key marketing appeal of e-cigarettes is the promise of avoiding harmful chemicals from combustion present in tobacco cigarettes. Despite these claims, vaping involves the inhalation of aerosols that may contain toxic compounds, such as formaldehyde, acetaldehyde, acrolein, and heavy metals, together with addictive substances, such as nicotine and cannabis.^(1-4)^ Additionally, while some e-cigarette users may believe they are engaging in a risk-free activity when smoking non-combustible products, recent studies have shown that vaporized liquids contain toxic compounds, such as pro flavors.^(5)^Moreover, most users are not aware of the mixed content in pods,^(6,7)^ especially when e-liquids are acquired from non-trustworthy or non-regulated sources.

Recently, research on the association between vaping and mental health has gained attention. Increased rates of depression and suicidality were found among vaping users.^(8,9)^A survey conducted with 2,500 teenagers and young adults showed that 60% of nicotine-only, THC-only, and dual vapers reported anxiety symptoms, as compared to 40% of participants who never vaped.^(10)^Taylor et al. found that smoking, vaping, and dual-use were more common among participants with a mental health condition than those without it.^(11)^Moreover, e-cigarette users are significantly more likely to have attention deficit hyperactivity disorder, post-traumatic stress disorder, gambling disorder, and anxiety, and report low self-esteem and impulsivity.^(12)^

Medical trainees may be particularly vulnerable to vaping-related disorders, not only because they fall within a high-risk young adult demographic but also owing to their high burden of mental health conditions.^(13-15)^ Compared with the general population, medical trainees exhibit higher rates of anxiety, depression, burnout, and suicidal ideation. These conditions frequently co-occur with addictive behaviors, although the temporal and causal relationships remain debated.^(14,16-18)^ Accordingly, our group previously demonstrated that the prevalence of e-cigarette use is much higher among medical trainees than in the general population in Brazil and the United States (US),^(19)^despite the increasing evidence of harm. Moreover, in these prior studies, medical trainees who vaped were more likely to report anxiety symptoms and concurrent substance use, including tobacco, marijuana, and alcohol, compared with their non-vaping counterparts.^(19)^

## OBJECTIVE

This study aimed to investigate whether the aforementioned mental health disorders differ between e-cigarette users with and without symptoms of dependence. Based on previous studies, we hypothesize that trainees with vaping-related disorders are more likely to have comorbid mental health conditions.

## METHODS

### Study design and participants

This study constitutes a secondary analysis of the Global Vaping Study (GVS). The GVS was a cross-sectional web-based survey conducted between October 2020 and November 2021. The research group’s main goal was to explore the knowledge, perception, and patterns of use of e-cigarettes and hookah among medical students, residents, and fellows. Data on e-cigarettes, hookah use, and knowledge about associated health risks have been reported elsewhere.^(19)^

We conducted the survey in Brazil (BR), the US, India (IN), Canada (CA), and the United Kingdom. To reach potential participants, the GVS researchers collaborated with academic associations, professors, deans, and students, using various channels, including social media, lectures, and mailing lists. We also promoted social media advertisements in the US and Brazil. In the US, participation was incentivized with a $50 gift card raffle. Details about the study support and funding can be found in the primary study article.^(19)^

Ethical approval was obtained from the ethics committees of leading institutions and medical schools that collaborated in the study’s recruitment efforts within their respective countries. All participants signed an informed consent form.

### Study questionnaire

The main study protocol and questionnaire were collaboratively developed by investigators from Brazil and the US in Portuguese and English. A comprehensive description of the study methods and questionnaire has been published elsewhere.^(19)^In the present analysis, we focused on questions about socio-demographics, vaping use and dependence, mental health conditions, and substance use.

### Sociodemographic variables

Age was reported as a discrete variable, whereas gender was classified into three distinct groups: female, male, and other/preferred not to answer. The level of education was stratified into two categories: medical school or residency/fellowship. Household income was categorized into four groups, corresponding to the income tax bracket strata in each country. Although social class is typically divided into five classes in Brazil, we grouped the lowest two classes into one to have four categories comparable to the US counterparts.

### Vaping use and dependence

Active vaping use was operationally defined as the consistent use of e-cigarettes at least once a month. Participants who had never vaped and those who had vaped previously but not at the time of survey completion were grouped into the category of non-active vaping users. Vaping dependence was assessed using the adapted Hooked on Nicotine Questionnaire for vaping,^(20)^a validated tool consisting of ten comprehensive questions. A positive screening result was determined if an individual reported experiencing two or more symptoms out of ten, indicating a level of dependency on vaping behavior.

### Mental health conditions and substance use disorders

Screening questionnaires were used to assess anxiety (GAD-2) and depressive symptoms (PHQ-2). Both were validated for the countries under study.^(21-23)^A positive screening result was defined as a score of three or higher on either questionnaire. Regarding psychiatric treatment, participants were divided into a group of past 12-month treatment and another with no treatment in the period. Finally, substance use (e-cigarette, traditional tobacco, hookah, marijuana) was queried in the form of an adapted version of the National Health Interview Survey.^(24)^ To identify unhealthy alcohol use, we incorporated four questions from the Alcohol Use Disorders Identification Test (AUDIT) instrument. The frequency of binge drinking was reported across five categories according to AUDIT (never, less than monthly, monthly, weekly, almost daily/daily). Alcohol use was subsequently categorized into two groups: those engaging in at least monthly binge drinking and those reporting less than monthly to no alcohol use.

### Statistical analysis

Participants were grouped into two categories based on the presence of dependence symptoms. Respondent characteristics were delineated for each country, with continuous variables reported as medians and interquartile ranges, and categorical variables reported as absolute and relative frequencies. We compared characteristics between participants with and without vaping dependence using χ^2^ tests for categorical variables and t-tests for continuous variables. We used a multilevel logistic regression to investigate the associations between mental health conditions, including anxiety symptoms, psychiatric treatment in the last year, past-month binge drinking, past-month tobacco smoking, and vaping dependence. We adjusted the analysis for age and gender. Country was modeled as a random effect to account for structural and regulatory differences between Brazil and the US. Depressive symptoms and marijuana use in the past month were not included in the regression, given that they did not achieve statistical significance in hypothesis testing. A significance level of 0.05 was considered acceptable. All analyses were performed in Stata version 14.2 (StataCorp, College Station, TX).

## ❚RESULTS

Of the 7,526 respondents to the GVS, 946 (15.3%) were classified as active e-cigarette users and constituted the analytic sample for this secondary analysis. Table 1 summarizes the characteristics of active vaping users in each country. Most participants were female (BR=57.0%, US=60.8%) and medical students (BR=91.8%, US=85.0%). There was a discrepancy in household income distribution, with the upper class representing 70.0% in Brazil and 13.5% in the US. Among e-cigarette users in both countries, we found a high prevalence of tobacco cigarette smoking, active hookah use, marijuana smoking in the past month, and binge drinking. However, a much higher prevalence of vaping-related disorder was found in the US cohort (BR=36.8%, US=92.5%, p<0.01). Participants from Brazil had more depressive symptoms (BR=38.1%, US=29.0%, p<0.01) but lower anxiety symptoms than their American counterparts (BR=17.8%, US=29.6%, p<0.01). More participants from the US reported psychiatric treatment in the previous year than their Brazilian counterparts (BR=30.9% US=53.6%, p<0.01).

**Table 1:** Characteristics of medical trainees with current vaping status according to country

Variables	Brazil (n=612)	US (n=334)
Age (years), median (IQR)^†^	22 (21–24)	28 (26–32)
Gender, n (%)		
Male	260 (42.5)	129 (38.6)
Female	349 (57.0)	203 (60.8)
Other/participant chose not to answer	3 (0.5)	2 (0.6)
Level of education		
Medical students, n (%)	562 (91.8)	284 (85.0)
Residents/Fellows, n (%)	50 (8.2)	50 (15.0)
Household income^†§^, n (%)		
Lower	10 (1.7)	34 (10.2)
Lower middle	61 (10.0)	126 (37.7)
Middle	111 (18.3)	129 (38.6)
Upper	425 (70.0)	45 (13.5)
Current hookah user	189 (30.1)	88 (26.3)
Tobacco smoking in the last month^†^, n (%)	293 (47.9)	272 (81.4)
Marijuana use in the last month^†^, n (%)	246 (40.2)	116 (34.7)
Frequency of binge drinking ≥ monthly, n (%)	499 (81.5)	181 (54.2)
Positive screening for depressive symptoms^✧^, n (%)	233 (38.1)	97 (29.0)
Positive screening for anxiety symptoms^¶^, n (%)	109 (17.8)	99 (29.6)
Positive screening for vaping dependence^†‡^, n (%)	225 (36.8)	309 (92.5)
Psychiatric treatment in the last year	189 (30.9)	179 (53.6)

^†^ There were five missing datapoints for age, five for household income, and 50 for vaping dependence status; ^§^ The following thresholds were adopted to classify household income in each country: Brazil (adapted from the Brazilian Institute of Geography and Statistics classification): <2 minimum wages per month, 2 to 5 minimum wages per month, 5 to 10 minimum wages per month, >ten minimum wages per month; US (Consolidated brackets based on data from the US Census Bureau): <US$50,000 per year, $50,000 to $80,000 per year, $80,000 to $100,000 per year, >$100,000 per year; ^✧^ PHQ2 score ≥3; ^¶^ GAD2 score ≥3; ^‡^ Hooked on Nicotine score adapted for vaping dependence ≥2.

When contrasting e-cigarette users with and without criteria for vaping use disorder, we found that those with a positive screening for dependence symptoms were older and frequently male. While there was no significant difference in the proportion of hookah or marijuana use, those with vaping use disorder were more likely to use tobacco in the previous month, present positive screening for anxiety, and seek psychiatric treatment in the previous year (Table 2). Binge drinking was highly prevalent in both groups, although significantly higher among e-cigarette users not reporting symptoms of dependence.

**Table 2:** Characteristics of medical trainees with current vaping status according to vaping dependence

Variables	No dependence^†‡^ (n=362)	Dependence^†‡^ (n=534)	p value
Age (years), median (IQR)^†^	23 (21–25)	25 (22–29)	<0.01
Gender, n (%)			
Male	195 (53.9)	329 (61.6)	0.02
Female/Other	167 (46.1)	205 (38.4)	
Level of education			
Medical students, n (%)	335 (92.5)	467 (87.5)	0.01
Residents/Fellows, n (%)	27 (7.5)	67 (12.5)	
Household income^†§^, n (%)			
Middle/Lower Middle/Lower	135 (38.3)	325 (58.0)	<0.01
Upper	227 (62.7)	209 (42.0)	
Current hookah user	112 (30.9)	141 (26.4)	0.13
Tobacco smoking in the last month^†^, n (%)	173 (47.8)	368 (68.9)	<0.01
Marijuana use in the last month^†^, n (%)	136 (37.6)	208 (39.0)	0.67
Frequency of binge drinking ≥monthly, n (%)	327 (90.3)	472 (88.4)	0.35
Positive screening for depressive symptoms^✧^, n (%)	116 (32.0)	195 (36.5)	0.16
Positive screening for anxiety symptoms^¶^, n (%)	63 (17.4)	138 (25.8)	<0.01
Psychiatric treatment in the last year	103 (28.5)	251 (47.0)	<0.01

^†^ There were five missing datapoints for age and 50 for dependence status; ^§^ The following thresholds were adopted to classify household income in each country: Brazil (adapted from the Brazilian Institute of Geography and Statistics classification): <2 minimum wages per month, 2 to 5 minimum wages per month, 5 to 10 minimum wages per month, >10 minimum wages per month; US (Consolidated brackets based on data from the US Census Bureau): <US$50,000 per year, $50,000 to $80,000 per year, $80,000 to $100,000 per year, >$100,000 per year; ^✧^ PHQ2 score ≥3; ^¶^ GAD2 score ≥3; ^‡^ Hooked on Nicotine score adapted for vaping dependence ≥2.

In the multilevel regression analysis, all variables except age were dichotomic. We found that psychiatric treatment in the previous year (OR=1.56, 95%CI=1.10–2.22) and male gender (OR=1.46, 95%CI=1.05–2.04) were associated with vaping dependence (Table 3). No association was observed for other variables, including mood symptoms or substance use.

**Table 3:** Multilevel regression analysis showing an association between participants’ characteristics and vaping dependence

Variables	Odds ratio	95% CI	p value
Age	1.02	0.98; 1.06	0.31
Household income^†^ (upper/other brackets)	1.41	0.98; 2.04	0.06
Gender (male/non-male)	1.46	1.05; 2.04	0.02
Tobacco cigarette use in the last month (yes/no)	1.20	0.87; 1.67	0.25
Positive screening for anxiety symptoms^§^, (yes/no)	1.5	0.76; 1.74	0.50
Psychiatric treatment in the last year, (yes/no)	1.56	1.10; 2.22	0.01

^†^ The following thresholds were adopted to classify household income in each country: Brazil (adapted from the Brazilian Institute of Geography and Statistics classification): <2 minimum wages per month, 2 to 5 minimum wages per month, 5 to 10 minimum wages per month, >10 minimum wages per month; US (Consolidated brackets based on data from the US Census Bureau): <US$50,000 per year, $50,000 to $80,000 per year, $80,000 to $100,000 per year, >$100,000 per year. In this analysis, the upper class was compared to the other brackets; ^§^ GAD2 score ≥3.

## DISCUSSION

This multicenter study examined vaping dependence and its association with mental health indicators among medical trainees from Brazil and the US. We observed a notably high prevalence of vaping dependence among active users compared with prior studies in general adolescent and young adult populations, highlighting vaping dependence as a relevant behavioral health concern within this population.^(20,25)^

Medical trainees experience sustained academic pressure, long working hours, and competitive environments, factors consistently associated with increased vulnerability to mental health symptoms and maladaptive coping behaviors. Consistent with prior findings from our group, vaping among trainees may function as a coping or social behavior.^(19)^However, the cross-sectional design precludes causal inference regarding the directionality between vaping and mental health outcomes.

Many e-cigarette users in our study use vaping as a nicotine delivery system, which could potentially exacerbate mental health conditions over time. In that sense, there is emerging concern that vaping may lead to the development and/or worsening of mood symptoms and substance use disorders in a vicious cycle.^(26,27)^Interestingly, a US national surveillance study found a graded association between vaping frequency and depression.^(28)^ Other studies showed that individuals with chronic mental disorders may rely on vaping as a mental health coping strategy.^(29-31)^Additionally, e-cigarette use can also increase the initiation of conventional cigarettes.^(19, 32)^

While previous studies have suggested associations between vaping frequency and depressive or anxiety symptoms, our multivariable analysis did not demonstrate independent associations between vaping dependence and current anxiety or depressive symptoms. These findings underscore the need for longitudinal studies to clarify temporality and identify potential confounders.

We found a significant association between prior psychiatric treatment and vaping dependence. Although causality cannot be inferred, this finding suggests that vaping dependence may signal broader mental health vulnerability. Accordingly, e-cigarette use and dependence should be considered red flags warranting comprehensive mental health screening and assessment of co-occurring risky behaviors.

Male gender was independently associated with higher odds of vaping dependence, consistent with established sex differences in substance use patterns. Higher income showed a marginal association (p=0.06), which may reflect greater access to and affordability of vaping products, although this finding should be interpreted with caution given the income categorization.

Finally, our results have significant implications for medical education and public health policies. There has been a heated debate on whether e-cigarettes can be used as tools for tobacco smoking cessation, with medical societies and public health agencies sometimes issuing conflicting recommendations.^(33,34)^The situation is more complex in Brazil, where sales of e-cigarettes have been prohibited since 2009, although illegal sales continue to increase over the years. Our group previously showed that medical trainees are currently ill-equipped to discuss vaping with their patients and lack basic knowledge of the health risks attributed to vaping, in addition to local marketing and sales regulations. Contextually, individuals may initiate vaping because they perceive it as harmless and not leading to dependence, unlike tobacco cigarettes. Regardless of its biological and/or social causes, vaping has a high addictive potential, and substance addiction has various negative implications. It is our view that this message should be reinforced in medical schools and to the public.

Although this cross-sectional study cannot establish causality, the high prevalence of vaping dependence among medical trainees underscores the need for coordinated action across sectors. Regulatory agencies should take a more proactive role in overseeing the production and commercialization of e-cigarette products. For example, Juul Labs has faced several lawsuits and reached litigation settlements worth billions of dollars, due to inadequate/missing hazard warnings, negligence, and intentional misrepresentation.^(35)^Medical societies play an important role in providing clear, evidence-based educational resources for patients, particularly in the context of widespread and often conflicting online information. Although integrating vaping-related content into formal curricula would be ideal, a more rapid and feasible approach is the use of practical toolkits that include screening strategies and brief educational interventions to prevent and address vaping-related disorders.^(36)^ Finally, healthcare providers need to be better equipped to identify and inquire about vaping-related disorders, which are increasingly common among medical trainees. Rather than adopting a paternalistic approach that dictates whether to vape, the focus should be on engaging in open and honest conversations about the associated cardiopulmonary and mental health risks based on the current literature.

### Limitations

This study had some limitations. First, the recruitment technique was based on a convenience sample, which may be subject to selection bias. Second, the reliance on self-reported data for sensitive domains such as substance use and mental health symptoms introduces the potential for social desirability bias. Even in anonymous surveys, participants may underreport behaviors or symptoms perceived as stigmatized or professionally undesirable, which could lead to conservative estimates of prevalence and associations.

## CONCLUSION

This is the first multicentric study, to the author’s best knowledge, on the association of mental health and vaping dependence among medical trainees. Although causal relationships cannot be established in this study, our findings suggest that vaping dependence is closely intertwined with mental health vulnerability. Vaping dependence was highly prevalent among medical trainees and significantly associated with male gender and a history of prior psychiatric treatment. Future longitudinal and cohort studies should investigate vaping patterns, product types, and mental health impacts, and evaluate the temporal association of vaping dependence and the prevalence of mental health disorders. Medical schools, healthcare providers, and regulatory bodies should coordinate to address vaping dependence among medical trainees. While medical schools are not treatment providers, they can implement population-level education and screening initiatives that enable early identification of high-risk individuals and facilitate timely referral to specialized care, supporting large-scale prevention without replacing clinical services.

## SUPPLEMENTARY MATERIAL

Supplementary material
