# Supplementary material for: Vaping and mental health disorders among medical trainees
Source: Einstein (Sao Paulo). 2026 Sep 18;24:eAO2193. doi: 10.31744/einstein_journal/2026AO2193 (PMC13682770; doi:10.31744/einstein_journal/2026AO2193)
Supplement: Supplementary file 1 [file 2317-6385-eins-24-eAO2193-suppl01.pdf]

SUPPLEMENTARY MATERIAL

Vaping and mental health disorders among medical trainees

Fernanda Gushken, Luiza Helena Degani-Costa, Fernando Pereira Bruno, Claudia Szlejf, Ana Bresser Tokeshi, Yasmin Fakhari Tehrani, Daniel Kaufman, Kandipudi K L Prasad, Pentapati SS Kumar, Limalemla Jamir, Matthew GK Benesch, Morag G Ryan, Hardeep Lotay, Jonathan P Fuld, Thiago Marques Fidalgo, on behalf of the Global Vaping Study investigators

DOI: 10.31744/einstein_journal/2026AO2193

Table 1S. Global vaping study collaborators

Brazil	
**Institution**	**Collaborators**
Hospital Israelita Albert Einstein (São Paulo, São Paulo)	Luiza Helena Degani-Costa (PI)<br>Fernanda Gushken<br>Claudia Szlejf<br>João Roberto Resende Fernandes
Universidade Federal de São Paulo / Escola Paulista de Medicina – Department of Psychiatry (São Paulo, São Paulo)	Thiago Marques Fidalgo (PI)<br>Luiza Helena Degani-Costa<br>Fernando Pereira Bruno<br>Ana Bresser Tokeshi<br>Christian Douradinho
UFOP - Universidade Federal de Ouro Preto (Ouro Preto, Minas Gerais)	Paulo César Rodrigues Pinto Corrêa
Univás - Universidade do Vale do Sapucaí (Pouso Alegre, Minas Gerais)	Antonio Carlos Aguiar Brandão
UNIDEP - Centro Universitário de Pato Branco (Pato Branco, Paraná)	Vilson Geraldo Campos<br>Lara Gandolfo
UNITAU - Universidade de Taubaté (Taubaté, São Paulo)	Oscar César Pires
UNINOVE Vergueiro (São Paulo)	Ramon Andrade Bezerra De Mello
Universidade Federal do Rio Grande do Sul - UFRGS (Porto Alegre, Rio Grande do Sul)	Marli Maria Knorst
Universidade Federal Fluminense - UFF (Rio de Janeiro)	Carlos Leonardo Carvalho Pessôa
Faculdade de Ciências Médicas da Santa Casa de São Paulo (São Paulo)	Ricardo Golmia
Pontifícia Universidade Católica de São Paulo - PUC-SP (Sorocaba, São Paulo)	Guilherme Borges Gomes da Silva
Centro Universitário Faculdade de Medicina do ABC - FMABC (Santo André, São Paulo)	Karine Corcione Turke<br>Antônio Carlos Palandri Chagas<br>Carla Janice Baister Lantieri
Universidade Cidade de São Paulo - UNICID (São Paulo)	Yasmin Paz De Marchi
Faculdade de Medicina de Ribeirão Preto - FMRP-USP (Ribeirão Preto, São Paulo)	Daniel Lacerda Heringer
Pontifícia Universidade Católica de São Paulo - PUCCAMP (Campinas, São Paulo)	Bruna Granig Valente
Pontifícia Universidade Católica de São Paulo - PUCCAMP (Campinas, São Paulo)	Larissa Moreira David
Universidade Santo Amaro - UNISA (São Paulo)	Carolina Lacerda Souza
Centro Universitário de Volta Redonda - UniFOA (Volta Redonda, Rio de Janeiro)	Juliana Monteiro Ramos
Faculdade de Medicina de Valença - UniFAA (Valença, Rio de Janeiro)	Rafael Moura de Almeida
Universidade Federal do Amazonas - UFAM (Manaus, Amazonas)	Thainá Mendonça Bentes
Universidade Federal do Paraná - UFPR (Curitiba, Paraná)	Vinicius Ryu Kami
Universidade Federal de Juiz de Fora - UFJF (Juiz de Fora, Rio de Janeiro)	Giancarlo Lucchetti
Centro Universitário de Brasília - UNICEUB (Brasília, Distrito Federal)	Marina Toscano Silveira
Universidade Federal do Acre - UFAC (Rio Branco, Acre)	Arthur Somavila Barros
Universidade de Passo Fundo - UPF (Passo Fundo, Rio Grande do Sul)	Vicente Bigolin Hauli
Universidade Estácio de Sá (Rio de Janeiro)	Luis Fernando da Silva Bouzas<br>Antonia Worcman de Carvalho<br>Juliana Moreira Guerra<br>Gabriel Lima Benchimol
Demeter Research (São Paulo)	Lincoln Basílio Alves
Instituto Tocantinense Presidente Antônio Carlos - UNITPAC (Araguaína, Tocantins)	Victória Mourão Luz
Universidade Federal de Alagoas - UFAL (Maceió, Alagoas)	Caroline Meneses Resende
Universidade Estadual do Ceará - UECE (Fortaleza, Ceará)	Antonio Andrei da Silva Sena
Faculdade de Medicina Estácio de Canindé (Canindé, Ceará)	Ana Karoline Mendes Sales
Universidade de Brasília - UNB (Brasília, Distrito Federal)	Gabriel Victor Silva Pereira

continue...

...Continuation

Table 1S. Global vaping study collaborators

India	
**Institution**	**Collaborators**
All India Institute of Medical Sciences (Mangalagiri, Guwahati & Delhi)	Aditi Mohta<br>Pentapati SS Kumar<br>Limalemla Jamir
Andhra Medical college, Visakhapatnam (Andhra Pradesh)	Kandipudi K L Prasad (PI)<br>Devi Madhavi Bhimarasetty<br>Makineedi Rama Lakshmi
Deccan College of Medical Sciences (Hyderabad)	Rajesh Neeluri
CIMS (Chamarajanagar)	Mahesh V
B. J. Medical College & Sassoon Hospital (Pune)	Pradnya V. Shinde
GIMSR Medical College (Andhra Pradesh)	Radha Ramani Bonu
Vinayaka Missions Medical College and Hospital (Karaikal)	Chithra Boovaragasamy<br>Gnanamani Gnanasabai
Government Medical College (Patiala)	Simmi Oberoi<br>Aman Dev Singh
Veer Chandra Singh Garhwali Govt. Institute of Medical Science & Research (Uttarakhand)	Tumul Nandan
**United States**	
**Institution**	**Collaborators**
Touro College of Osteopathic Medicine (New York)	Fernando Pereira Bruno (PI)<br>Yasmin Fakhari Tehrani<br>Daniel Kaufman<br>Cameron Ghassemi
**Canada**	
**Institution**	**Collaborators**
Memorial University of Newfoundland (Newfoundland & Labrador)	Matthew GK Benesch (PI)<br>Morag G Ryan
**United Kingdom**	
**Institution**	**Collaborators**
University of Cambridge (Cambridge)	Jonathan P Fuld (PI)<br>Hardeep Lotay

The following collaborators are recognized as collaborating co-authors for their efforts in advertising and recruiting participants in Brazil, India, and the US.
